# Nanovectorized radiotherapy: a new strategy to induce anti-tumor immunity

**DOI:** 10.3389/fonc.2012.00136

**Published:** 2012-10-10

**Authors:** Claire Vanpouille-Box, François Hindré

**Affiliations:** ^1^LUNAM Université, Université d’AngersAngers, France; ^2^INSERM U1066 Micro et Nanomedecines BiomimétiquesAngers, France

**Keywords:** anti-tumor immunity, nanoparticle, radionuclides, biomaterials, active targeting

## Abstract

Recent experimental findings show that activation of the host immune system is required for the success of chemo- and radiotherapy. However, clinically apparent tumors have already developed multiple mechanisms to escape anti-tumor immunity. The fact that tumors are able to induce a state of tolerance and immunosuppression is a major obstacle in immunotherapy. Hence, there is an overwhelming need to develop new strategies that overcome this state of immune tolerance and induce an anti-tumor immune response both at primary and metastatic sites. Nanovectorized radiotherapy that combines ionizing radiation and nanodevices, is one strategy that could boost the quality and magnitude of an immune response in a predictable and designable fashion. The potential benefits of this emerging treatment may be based on the unique combination of immunostimulatory properties of nanoparticles with the ability of ionizing radiation to induce immunogenic tumor cell death. In this review, we will discuss available data and propose that the nanovectorized radiotherapy could be a powerful new strategy to induce anti-tumor immunity required for positive patient outcome.

## INTRODUCTION

The Janus face of the immune system in carcinogenesis has long been controversial and one of the most challenging in immunology. With progress in biological tools such as transgenic mouse technologies, it is now recognized that the immune system plays a dual role in cancer. For instance, it suppress tumor progression by identifying and destroying neoplastic cells (Dunn et al., 2002; Schreiber et al., 2011) but also promotes tumor growth by selecting tumor cells more adept at evading immune-mediated destruction (Khong and Restifo, 2002; Smyth et al., 2006; Zitvogel et al., 2006; Vesely et al., 2011) leading to the establishment of an immunosuppressive microenvironment that fosters carcinogenesis (Radoja et al., 2000; Whiteside, 2008). However, the host immune system not only impacts on cancer development but also on response to treatment. Experimental evidence strongly supports the concept that the activation of the immune system is essential for successful chemo- and radiotherapy (Casares et al., 2005; Apetoh et al., 2007b; Obeid et al., 2007a,2007b; Zitvogel et al., 2008). By improving the quality of released signals, some conventional treatments trigger a peculiar type of cell death that elicits a potent anti-tumor immune response required for positive patient outcome (Zitvogel et al., 2008). Called “immunogenic cell death” (ICD), this type of tumor cell death is defined by at least three signals: calreticulin (CRT) exposure (Obeid et al., 2007b; Zitvogel et al., 2010), release of high mobility group box-1 (HMGB-1; Apetoh et al., 2007a,2007b), and ATP (Ghiringhelli et al., 2009; Martins et al., 2012). Among all current available treatments, only radiotherapy (Chakraborty et al., 2004), anthracyclines (Casares et al., 2005; Mattarollo et al., 2011), oxaliplatin (Panaretakis et al., 2009; Tesniere et al., 2010), and cyclophosphamide (Schiavoni et al., 2011) have been shown to generate these signals in the proper spatiotemporal order leading to an *in situ* tumor vaccine (Ma et al., 2010; Hannani et al., 2011).

Therefore, conventional treatments could be used not only for their cytocidal effects but also for their ability to induce anti-tumor immunity. This idea extends far beyond treatments that already exhibit pro-immunogenic effects since envisioning the use of immune response modifiers (IRM) to optimize the synergy with the immune system offers great opportunities to provide alternative ways of tumor-specific immunity (Schiller et al., 2006; Cheever et al., 2008). For instance, Demaria and colleagues demonstrated significant increase in treatment efficiency when radiotherapy is combined with anti-cytotoxic T lymphocyte antigen-4 (CTLA-4; Demaria et al., 2005; Matsumura et al., 2008; Dewan et al., 2009; Pilones et al., 2009), a monoclonal antibody that blocks CTLA-4 receptor well-known to be implicated in immune tolerance (Peggs et al., 2006; O’Day et al., 2007).

In consideration of this emerging vision, the ability of anti-cancer strategies to induce anti-tumor immunity has to be investigated. Among new treatment approaches, internal radiotherapy using nanoparticles (NPs) holds great promise for the management of refractory tumors (Allard et al., 2008; Vanpouille-Box et al., 2011b). Primarily designed to focus radiation to a specific target while protecting healthy tissues from radiation, nanovectorized radiotherapy has been shown to elicit anti-tumor immunity in a preclinical model of glioblastoma (Vanpouille-Box et al., 2011a). This new treatment concept is based on the use of NPs as reservoir for radionuclides enabling the entrapment of alpha (α) and beta (β) emitters conferring them different ways to directly kill tumor cells as well as distinct interactions with the microenvironment (Ting et al., 2010). The NP itself can also be designed to have properties of an IRM able to modify and improve the immune response through the use of peculiar biomaterials and/or surface ligands. Therefore, nanovectorized radiotherapy that combines ionizing radiation and nanodevices, is one therapy that could boost the quality and magnitude of an immune response in a predictable and designable fashion. Given the novelty of nanomedicines application, only a few studies analyzed NP’s adjuvant effect on the host’s innate and adaptive immune response. In this review, we will discuss available data and propose that the nanovectorized radiotherapy could be a powerful new strategy to induce anti-tumor immunity required for successful anti-cancer treatment.

## NANOPARTICLE: A NEW KIND OF IMMUNE RESPONSE MODIFIER

**FIGURE 1:**
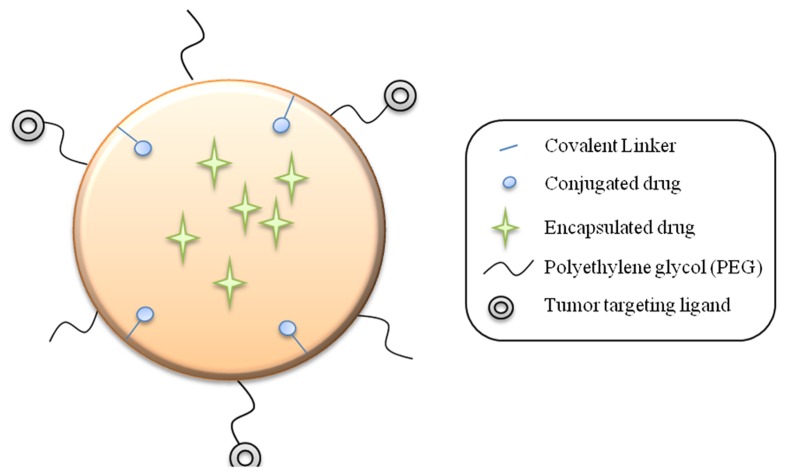
**Schematic nanoparticle**.

The ideal anti-cancer treatment would be the one capable of reducing and eliminating tumors without causing any damage to surrounding healthy tissues. In that context, over the past two decades, nanotechnology-based approaches have emerged as a promising field that aims at overcoming limitations encountered in conventional anti-cancer treatments. Numerous nanodevices have been engineered using top-down or bottom-up approaches, generally ranging in dimensions from one to a few hundred nanometers in at least one dimension (Perry et al., 2011). NPs can be designed to carry therapeutics drugs (chemo- or radio-therapeutics) loaded on or within the nanocarriers by chemical conjugation or simply by encapsulation (**Figure 1**; Sengupta et al., 2005; Vanpouille-Box et al., 2011b; Vrignaud et al., 2011). Therefore, NPs have the ability to improve stability of encapsulated drug as compared to free entities and release in a more controlled manner over time to maintain anti-cancer agents within a therapeutic window (Amstad and Reimhult, 2012). Additionally, their flexible chemical properties allow NP surface modifications to increase their blood circulation half-life and improve their biodistribution profile. For instance, NP can be functionalized with polyethylene glycol (PEG) in order to generate a steric barrier on the surface preventing adherence of opsonins to the NP and therefore reducing their clearance by the reticuloendothelial system (RES; Otsuka et al., 2003; Yoncheva et al., 2005).

A wide range of nanodelivery systems are currently in development. NPs can be composed of natural (Liu et al., 2011; Tavangar et al., 2011) or synthetic (Powell et al., 2011), and degradable (Huynh et al., 2009) or non-degradable polymers (Peek et al., 2008). The choice of components that constitute the nanodevice is critical as it considerably influence the NPs properties. For instance, the drug release profile can be tuned by the size and material composition of the NP (Paillard et al., 2010). Additionally, the NP is amenable to surface modifications (Brannon-Peppas and Blanchette, 2004; Fahmy et al., 2005; Weiss et al., 2007; Beduneau et al., 2008; Byrne et al., 2008; Hirsjarvi et al., 2011; Talekar et al., 2011) providing them targeting properties to reach specifically an organ or even a specific cell (Weissleder et al., 2005; Beduneau et al., 2008; Gu et al., 2008; Talekar et al., 2011). With this unique ability, NPs can easily be engineered to precisely synergize with the immune system and be considered as a powerful “smart” IRM designed to reach a specific location and to interact with specific cells.

As a result, we will discuss each steps that could be harnessed in NP’s designing to interact with the immune system in a predictable fashion, that are (1) the choice of biomaterials that composed the NPs, (2) the proper size and charge of NPs to better synergize with the host, and (3) the possible use of ligand on NPs surface to specifically target immune or tumor cells.

### IMMUNE ADJUVANT PROPERTIES OF NANOPARTICLES COMPONENTS

The main goal of immunotherapy-based strategy is to harness immune system not only to fight cancer by targeting and killing tumor cells in a specific manner, but also to alert the immune system so that the residual tumor cells are kept in check. Active forms of immunotherapy, including cancer vaccines, represent one of the promising strategies. These approaches aims at inducing the activation and expansion of tumor-specific T cells, which have proven to be the most powerful immune mechanism to clear tumors (Porter et al., 2011).

**FIGURE 2:**
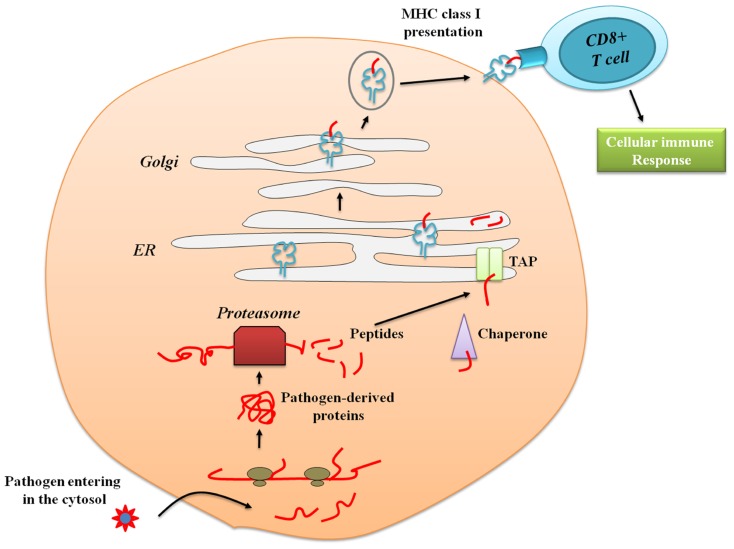
**Antigen presentation via major histocompatibility complex class I.** Pathogen-derived or self-proteins within the cytosol of antigen presenting cells (APCs) are enzymatically digested into peptides, mainly by cytosolic proteases (proteasomes), and are then transported by transporter associated with antigen processing molecules (TAP) into the endoplasmic reticulum (ER). In the ER lumen, peptides bind to MHC class I molecules, which are subsequently transported via the Golgi to the plasma membrane. The endogenous antigen presented by MHC class I will then be recognized by the CD8^+^ T cells leading to adaptive cellular immune responses.

**FIGURE 3:**
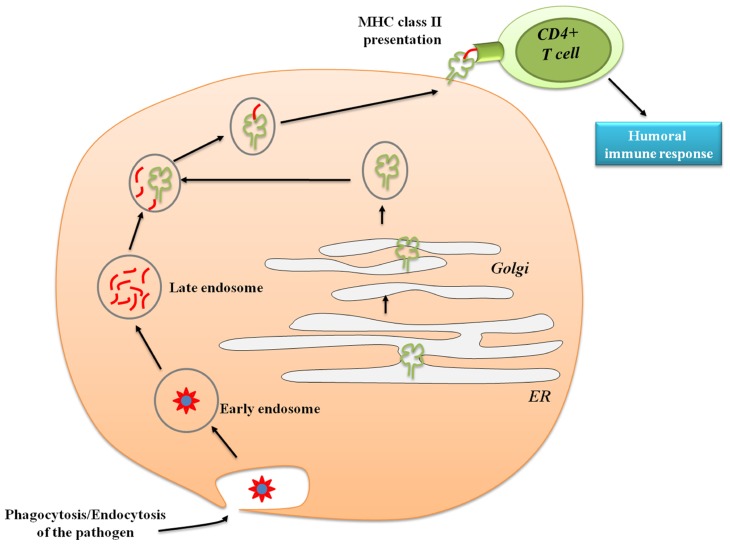
**Antigen presentation via major histocompatibility complex class II**. Exogenous antigens are derived from proteins that are endocytosed and processed by proteases. Peptides bind to newly synthesized MHC class II molecules in specialized antigen-processing vesicles (MHC class II-enriched compartment), and the complexes are externalized to the plasma membrane. CD4^+^ T cells will then recognize the exogenous antigen presented by MHC class II leading to the stimulation of CD4 T cells humoral responses.

Many efforts have focused on enhancing cross-presentation, a process mediated by antigen presenting cells (APCs) that are defined as cells that can process antigens of both endogenous and exogenous origin (Trombetta and Mellman, 2005). Endogenous antigen (such as normal cell proteins, tumor or viral antigens) are processed in the cytosol and presented in the context of major histocompatibility complex (MHC) class I molecules to be recognized by CD8^+^ T cells (**Figure 2**; Itano and Jenkins, 2003) leading to strong cytolytic and Th1 inflammatory responses. APCs are also capable to internalize exogenous antigens. The latter are processed in specialized compartments called endocytic vesicles or endosome, and presented through MHC class II molecules to be recognized by CD4^+^ T cells (**Figure 3**; Watts, 2004). APCs include B cells, macrophages, and dendritic cells (DC). Because of their wide distribution, location at critical sentinel sites (skin and mucosal surfaces), intrinsic migratory capacity, and ability to activate naïve T cells, DCs are considered as the most powerful professional APCs (Itano and Jenkins, 2003; Trombetta and Mellman, 2005; Delamarre and Mellman, 2011). DCs are indeed capable of processing both exogenous and endogenous antigens and present peptide in the context of either MHC class I or II molecules. As DCs mature, they acquire the properties necessary to form and transport peptide-loaded MHC class II complexes to the cell surface (Cella et al., 1997). Antigen transport to the cell surface is correlated with increased expression of co-stimulatory molecules, such as CD80, CD86, and CD40, molecules well-known to amplify T cell receptor (TCR) signaling and promote T cell activation (Ni and O’Neill, 1997). Given the critical role of DC in eliciting adaptive immune response, efforts have been made to develop new strategies that target and stimulate DCs.

Nanomedicine-based treatments represent one of the main promising approaches since nanoscale drug delivery system could be thought and designed from the beginning to properly interact with the host immune system. For instance, some NPs are able to entrap drug already known to induce ICD (i.e., radionuclide; Sun and Xie, 2011; Tang et al., 2011; Vanpouille-Box et al., 2011a), oxaliplatin (Jain et al., 2010; Paraskar et al., 2012), and cyclophosphamide (Salgueiro et al., 1999), and exhibit biological effects such as endolysosomal escape (Panyam et al., 2002; Paillard et al., 2010) and biological barrier crossing (De Jong and Borm, 2008; Paillard et al., 2010). Among them, NPs of poly(D,L-lactide-co-glycolide) (PLGA) hold great promise and have been extensively studied for their ability to activate DCs for priming antigen-specific T cell responses (reviewed in Hamdy et al., 2011). PLGA is a Food and Drug Administration (FDA)-approved biodegradable polymer that had been widely used in several controlled release drug products for human use (Jain, 2000; Dinarvand et al., 2011; Jain et al., 2011). One of the main characteristic of PLGA relies with its flexibility that allows manipulating its physico-chemical properties. Therefore, PLGA can shift the delivery of encapsulated drugs to either cytoplasm (for MHC class I presentation and CD8^+^ T cell activation) or to the endosome (for MHC class II and CD4^+^ T cells activation; Hamdy et al., 2007,^2008^; Heit et al., 2007). More recently, PLGA has been shown to activate the NOD-like receptor family pyrin domain containing 3 [NLRP3 also known as cryopyrin, cold-induced autoinflammatory syndrome 1 (CIAS1) or NALP3] inflammasome (Demento et al., 2009; Sharp et al., 2009). It has indeed been demonstrated that cellular internalization of PLGA and polystyrene microparticles activate of the NLRP3 inflammasome through lysosomal damage and caspase-1 activation leading to the production of large amount of IL-1β by DCs (Sharp et al., 2009). The ability of NP’s components to directly influence NLRP3 inflammasome is very important since it has been described that NLRP3 inflammasome and subsequent IL-1β secretion is critical for stimulation of anti-tumor T cells responses following chemotherapy (Ghiringhelli et al., 2009; Menu and Vince, 2011).

Poly(D,L-lactide-co-glycolide) is not the only strategy that has been investigated to achieve DC cross-presentation. The use of pH-responsive materials that naturally foster antigen escape from the endosome into the cytosol where MHC class I antigen processing begins has emerged. For instance, Murthy et al. (1999) and Jones et al. (2003) have developed synthetic polymer containing alkyl(acrylic acid) monomers that become protonated at endosomal pH levels (5.5–6.5). Once protonated, the polymers destabilize the endosomal membrane and allow antigen to escape into the cytoplasm (Jones et al., 2003).

Other particle materials can stimulate signaling pathways that lead to cellular activation. Baba and colleagues have shown that poly(gamma-glutamic acid) NPs can be used as a vaccine adjuvant. These NPs induced DC maturation through MyD88-mediated nuclear factor kappa B (NF-κB) activation and the p38 mitogen-activated protein kinase (MAPK) pathways, in a manner somewhat similar to lipopolysaccharide (LPS)-induced maturation of DC (Uto et al., 2007,2011a,2011b; Hamasaki et al., 2010). Therefore, NPs components act as immune adjuvant simply by inducing maturation of DC. This concept was also supported by Babensee and colleagues and Elamanchili and colleagues work, showing that exposure of bone marrow derived DC to polymers, notably PLGA, results in DC maturation as measured by the up-regulation of cell surface stimulatory markers such as MHC class II, CD40, CD80 and CD86 (Diwan et al., 2003; Elamanchili et al., 2004; Yoshida and Babensee, 2004,^2006^; Yoshida et al., 2007; Babensee, 2008).

Taken together, evidences clearly indicate that nanodevices for targeted delivery of drugs or radionuclides can be composed of biomaterials that possess different immune adjuvant properties. Therefore, the choice of biomaterials to design NPs could provide a potent tool to induce anti-tumor immunity.

### INFLUENCE OF NANOPARTICLE SIZE AND CHARGE ON IMMUNE SYSTEM

Another parameter to consider for immunogenic NP designing is the size and the charge of the NP. DCs and macrophages are both phagocytic cells. Hence, particles with dimension similar to pathogens (≥10 μm) are generally readily phagocytosed. Studies have shown that DCs preferentially phagocyte smaller particles in the viral range, while macrophages more efficiently ingest bacterial size particle (Gamvrellis et al., 2004). It has also been reported that NP with a diameter <500 nm were more effective in stimulating cytotoxic T lymphocytes (CTL) responses *in vivo* (Allsopp et al., 1996; Nixon et al., 1996). Possible explanation relies with the interactions of NPs with opsonins. Indeed, larger surface area of the NP allows more opsonins bounding and therefore, a faster degradation and rapid release of the encapsulated drug inside the phagosome (Owens and Peppas, 2006).

Additionally, physico-chemical properties of particle surface, particularly surface charge and surface chemistry, are known to affect both DC uptake and maturation. For instance, positively charged cationic particles in general have greater initial affinity toward cell surface than negatively charged or neutral par- ticles (Josephson et al., 1999; Foged et al., 2005; Perez-Martinez et al., 2011).

### SURFACE MODIFICATION OF NANOPARTICLES

To promote and enhance specific interactions between NP and the microenvironment, the surface of particles can be decorated with targeting moieties that are recognized specifically by targeted cells. Two main strategies can be envisioning: the one that target immune cells and the other one that target tumor cells to kills them and therefore, to provide proper “danger signal” required for immune system activation.

#### Immune cells targeting

In order to specifically enhance the maturation of DC, Palumbo et al. (2011) bound CD40 ligand (CD40L) on NP’s surface. CD40L is indeed transiently expressed on activated CD4^+^ T helper cells and its binding with the CD40 receptor on DCs is important for their complete maturation and transformation into competent APC (Loskog and Totterman, 2007). However, no significant results have been reported in their studies, suggesting the complexity of conferring immunogenic properties to NPs (Palumbo et al., 2011).

In another study, Dominguez and Lustgarten (2010) engineered immunogenic NPs to induce anti-tumor immune response. They indeed succeeded in binding not only one ligand but two to further stimulate the immune system. By linking anti-neu mAb directed against a tumor antigen and anti-CD40 mAb on NP’s surface, they generated an anti-tumor response resulting in tumor rejection with high production of Th1-proinflammatory cytokines, a stark reduction of regulatory T cells within the tumor and activation of specific cytotoxic immune response (Dominguez and Lustgarten, 2010). These recent results strongly support the potential use of biodegradable NPs to stimulate a tumor-specific immune response.

#### Tumor cell targeting

Specific tumor targeting could indirectly stimulate the immune system if the quantity and the quality of released signal in a specific location (i.e., the tumor) can be achieved. Many active targeting of NPs to tumor has been extensively studied and led notably to the development of NP conjugated with specific ligands that recognize a tumor-surface marker.

Over the past three decades, the generation of murine mAbs against tumor-associated antigens became a focal point of research illustrated by numerous studies being reported during the 1980s that dealt with NPs and mAb binding to their surface (Leserman et al., 1980; Barbet et al., 1981; Harsch et al., 1981; Hashimoto et al., 1983; Guidoni et al., 1984). Since then, a number of clinical trials have demonstrated the feasibility of antibody-based targeting (Bernard-Marty et al., 2006; Yoong et al., 2011; Foran, 2012; Smyth and Cunningham, 2012). Among mAb that were studied, Trastuzumab (or Herceptin®), a mAb that binds to the human epithelial growth factor receptor 2 (HER2), has been bound on NP’s surface to specifically target breast cancer cells (Hayes et al., 2006; Kirpotin et al., 2006). This targeting strategy has improved therapeutic efficiency of an HER2-targeted NPs formulation in comparison to its non-target one (Park et al., 2002).

Although antibodies have proven to be effective targeting agents, there are inherent issues such as decreased receptor affinity due to inadequate conjugation methods, insufficient tumor cell penetration, and non-specific binding of antibodies to cellular receptors. In that context, new technologies are currently being explored to enhance the selectivity and efficacy of ligands while attempting to overcome the shortcomings associated with existing targeting moiety. For example, peptides have recently emerged as targeting agent owing to the relative simplicity of synthesis and purification. The integrin family, particularly the αvβ3 integrins, has been widely studied to target cancer cells with NPs. For instance, a synthetic peptide of arginine–glycine–aspartic acid (RGD) residues has been used as a ligand conjugated to NPs for targeting αvβ3 integrins expressed on endothelial cells. Recent studies are further optimizing integrin targeting by engineering novel peptide moieties which bind with better affinity to integrins that current RGD tags (Ji et al., 2012; Xu et al., 2012; Zhan et al., 2012).

Binding bombesin (BBN) synthetic peptides on NP’s surface is another targeting strategy in development. BBN peptides are composed with 14 amino acids and present high affinity toward gastrin-releasing peptide (GRP) receptors (Smith et al., 2005) that are overexpressed in many cancer such as prostate (Markwalder and Reubi, 1999; Nagasaki et al., 2012), breast (Chao et al., 2009), and small-cell lung carcinoma (Moody et al., 1985; Oremek and Sapoutzis, 2003). Promising results were reported, notably by Chanda et al. (2009),^2010^, which demonstrated that the conjugation of BBN peptides on gold NPs’ surface lead to selective uptake of NP-BBN conjugates in prostate tumor sites.

However, NPs targeting strategies are not limited to those two approaches. Conferring targeting properties to NPs was indeed one of the main focuses of nanomedicine (Katsogiannou et al., 2011; Kolhatkar et al., 2011; Talekar et al., 2011). Therefore, a plethora of ways to generate “smart” NPs targeting a specific cell is currently in development which highlights the extreme flexibility of this new technology.

## RADIONUCLIDES FOR NANOVECTORIZED RADIOTHERAPY

Conventional radiotherapy (X-rays) is the mainstay adjuvant treatment of cancer. However, the radiation dose to surrounding normal tissues often limits its use and therefore, opened a very challenging research area in radiation oncology: the one that aims at reducing and destroying tumors without causing any damage to healthy tissues.

In that context, new external photon beam radiation therapy modalities have recently been emerged with the development of three-dimensional conformal radiotherapy (3D-CRT)/ volumetric-modulated arc therapy (VMAT), helical tomotherapy, intensity-modulated radiotherapy (IMRT), γ-rays (^60^Co)-knife-therapy, cyber-knife-radiotherapy–radiosurgery with 4D-image-guided tracking and 6D-image-guided stereotactic-radiotherapy, that dynamically synchronize imaging, patient positioning and treatment delivery with a dose escalation. These new approaches allow obtaining more conformal “radio-ablative” treatment of tumor lesions while minimizing the damage to the nearby normal tissues (Deb and Fielding, 2009; Teoh et al., 2011; Yu and Tang, 2011; Wen et al., 2012).

Another increasing successful radiation technique is the hadron therapy that uses a focus beam of quark-constituted of proton (H^+^), carbon ion or neutrons, allowing more precise ionizing radiation delivery. Compared to photons (X-rays and γ-emissions), proton beams are characterized by a low entrance dose while a maximal at a user-defined depth (“Bragg peak”) and almost no damage on the exit path. As a result, the chief advantage of proton therapy relies with its ability to precisely localize the radiation dosage compared to other form of external beam radiotherapy (DeLaney, 2011; Liu and Chang, 2011).

These newly developed external either photon- or especially hadron-therapy technologies are becoming more and more competitive, as for precisely target locally confined tumors, with brachytherapy modalities as alternatives options to anyhow carried out surgical approaches.

Radiation brachytherapy with either permanent interstitial implantation or temporary implant has also gained large acceptance in the last decades particularly for the management of prostate cancer (Alberti, 2011; Gomez-Veiga et al., 2012) and cervical cancer (Beddy et al., 2011; Walsh et al., 2011). This internal radiation approach is highly linked to the tumor type and size. For instance, brachytherapy is usually initiated toward the ends of external beam radiation after tumor regression has occurred and allows high doses to be delivered to the residual disease with relative sparing of surrounding normal tissues (Monk et al., 2007).

Another arm of brachytherapy consists in harnessing nanomedicines, such as radiolabeled monoclonal antibodies and/or biomaterial vectors, to generate a localized radiation (Allard et al., 2008; Barbet et al., 2012; Memon et al., 2013). As a result, with the identification of biological target overexpressed in cancer, brachytherapy is no longer limited to a specific tumor. In that context, nanovectorized radiotherapy that combines NPs and ionizing radiation is becoming a potent new radiotherapy approach that also overcomes non-specific radiation. Radioactive NPs have indeed been shown to modify the radiation distribution profile of a radionuclide by avoiding its fast elimination (Vanpouille-Box et al., 2011b) but also by maintaining radiation to a specific location for 96 h after their injection (Vanpouille-Box et al., 2011a). Even if few data regarding radioactive particle loading capacity, specific radioactivity has been shown to be compatible with clinic application (Salem et al., 2002,^2005^).

Compared to the newly developed radiotherapy strategies, nanovectorized radiotherapy presents the main advantage of being a low-cost technology by the use of radionuclides eluted from generators easily available, such as the ^188^W/^188^Re generator (Lepareur et al., 2011). More importantly, radioactive NPs’ formulation is simple providing them high availability and accessibility to patient. As a consequence, a spread of this new technology in most of clinical institutions, including those of developing countries, can be envisioned.

Radionuclides that decay by the following three general categories of decay have been studied for therapeutic potential of nanovectorized radiotherapy: beta (β)-particles emitters (yttrium-90, rhenium-188; Li et al., 2004; Tsai et al., 2011), alpha (α)-particles emitters (bismuth 213, astatine-211; Sofou et al., 2004; Couturier et al., 2005; Boskovitz et al., 2009) and auger electron-emitters (iodine-125, gallium-67; Snelling et al., 1995). However, the extreme toxicity of auger particles as well as concerns regarding radioprotection limited their use (Bodei et al., 2003; Milenic et al., 2004). Therefore, we will focus on α- and β-emitters and discuss their main characteristics that may lead to different interactions with the microenvironment.

### ALPHA (α) vs. BETA (β) EMITTERS

Particles emitted during atomic decay can be classified as low or high linear energy transfer (LET) radiation. The LET corresponds to the energy released by the radiation over a certain distance (expressed in keV/μm). At absorbed doses that are equivalent to those of low-LET radiation, high-LET particles are more cytotoxic. This phenomenon is called “radiation quality.” Most of the radionuclides used in internal radiotherapy; such as iodine-131 (Grunwald and Ezziddin, 2010; Leahy and Turner, 2011), yttrium-90 (Kulik et al., 2008; Menda et al., 2010; Kunikowska et al., 2011), lutetium-177 (Gains et al., 2011; Kunikowska et al., 2011), ^188^Re (Kumar et al., 2007; Torres-Garcia et al., 2008), or rhenium-186 (Syed et al., 2006; van Dodewaard-de Jong et al., 2011); emit low-LET radiation of 0.2 keV/μm in the form of β-particles as well as internal conversion electrons (Milenic et al., 2004). High-LET particle emitters used in internal radiotherapy only include the α-emitters bismuth-213, bismuth-212, and astatine-211, as well as lead-212 and actinium-225, which generate bismuth-212 and bismuth-213, respectively. These radioisotopes emit high-LET radiation (60–230 keV/μm) that produces clusters of DNA damage that are difficult to repair.

Linear energy transfer is intimately linked to the energy carried by a particle and the depth it penetrates into the biological tissue. Therefore, β-particles carry intermediate energy (0.50–2.30 MeV) but have a long range in tissues (1–12 mm of tissue penetration). This lengthy range reduces the need for cellular internalization and so targeting close to or at the cell membrane is sufficient. Additionally, the range of β-particles, as compared to the diameter of cells, allows them to traverse clusters of cells (from 10 to 1,000 cells; O’Donoghue et al., 1995).

Alpha-particles have a high energy (5–8 MeV) and an intermediate path length (50–80 μm) in biological tissues that corresponds to the diameter of several cells (2–10 cells).

Beta-emitters and alpha-emitters are produced either by cyclotron irradiation or by reactor irradiations, incorporated into a generator, and subsequently eluted (Haddad et al., 2008; Halime et al., 2009; Bakht and Sadeghi, 2011; Pillai et al., 2012). For therapeutic application, numerous criteria have to be considered while selecting a radionuclide. Therefore, regarding the tumor size, the advantage of a type of radiation decay will be preferably used in a specific application. For instance, β-particles will be more suitable radionuclides for solid tumors because of their ability to deposit a large amount of energy at a high dose rate. However, other criteria have to be considered for clinical applications: (1) availability of the radionuclide at a reasonable cost, (2) proper nuclear decay properties and absence of hindering daughter nuclides, and (3) a physical half-life long enough to allow internal radiotherapy. As a consequence, among all radionuclide available, only a few are currently developed for nanovectorized radiotherapy (Sofou et al., 2004; Allard et al., 2008; Hamoudeh et al., 2008; Bult et al., 2010; Vanpouille-Box et al., 2011a,2011b). Explanations can mainly be ascribed to the variable pertaining to their physico-chemical properties and to their chemistry that could be somewhat complex according to the NP used.

It is well-established that the radiobiology of high-LET radiation differs greatly from that of low-LET radiation (Goodhead et al., 1993). For instance, increase mRNA expression of inflammatory mediators and cytokines [e.g., interferon-γ (IFNγ)] that prompt immune responses has been identified in lymphocytes after their exposure to low-LET radiation (Amundson et al., 2000,^2004^; Kang et al., 2003). In this respect, we can suppose that APCs are able to detect radiolytic products that lead to the production of cytokines such as IFNγ, well-known to be implicated in adaptive immune response (Schoenborn and Wilson, 2007). An increased expression of genes coding for CD1C, CD1D, CD40, CD69, and IFNγ in lymphocytes after α-radiation exposure has been reported (Turtoi et al., 2010). Turtoi and Schneeweiss (2009) and Turtoi et al. (2010) indeed showed that a number of rapidly modulated early response genes in α-particle-irradiated lymphocytes that are associated with DNA repair and immune response mechanisms. However, the current knowledge of the biology of high-LET radiation is insufficient to make definite conclusions.

### EFFECT OF THE NANOVECTORIZED RADIOTHERAPY ON IMMUNE SYSTEM ACTIVATION

Immunotherapies are rarely effective as monotherapy but growing evidence supports a synergy between radiotherapy and IRM (Demaria et al., 2005; Dewan et al., 2009; Formenti and Demaria, 2009; Pilones et al., 2009; Newcomb et al., 2010). Among emerging new approaches, nanovectorized radiotherapy holds great promises as a new powerful anti-cancer treatment that could harness immunogenic properties of both NPs and ionizing radiations. Supporting this concept, we recently demonstrated that NPs loaded with rhenium-188, a β-emitter, are potent stimulators of tumor-specific immune response resulting in tumor rejection with high production of IFNγ cytokine, increase recruitment of immune effector T cells within the tumor and memory response in long-term survivor animals (Vanpouille-Box et al., 2011a). Intriguingly, remarkable survival benefit was only seen when two different types of stereotactic injections were used suggesting that the distribution of NP loaded with rhenium-188 within the tumor has a direct impact on the treatment efficiency. Therefore, the use of radionuclide within NP could provide additional advantages as compared to conventional radiotherapy where the distribution of ionizing radiation is homogenous.

**FIGURE 4:**
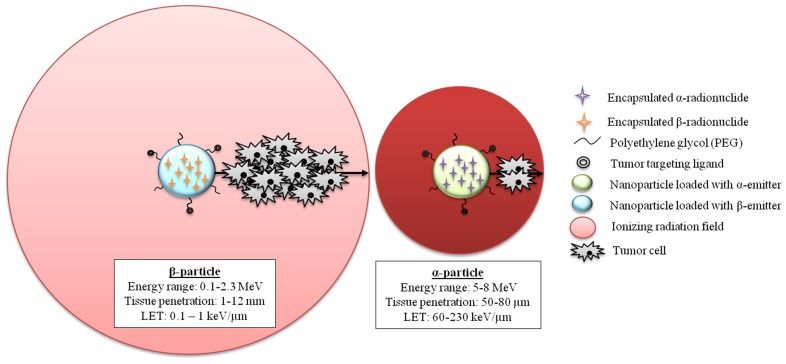
**Schematic depiction of nanoparticles loaded with beta- and alpha-emitters**.

Much work remains to be done to determine the effects of both low-LET (β-emitters) and high-LET (α-emitters) emitters on the host immune system. Nevertheless, the capability of NPs to entrap α- and β-radionuclides potentially provides additional means to fine tune the microenvironment interactions (**Figure 4**). Further investigations are required to better understand the interactions between ionizing radiations and the host immune system. Nevertheless, the potential benefits of nanovectorized radiotherapy may be based on the unique combination of immune-stimulatory NP with the ionizing radiation ability to induce an immunogenic tumor cell death.

## CONCLUSION

In summary, NPs represent a potent immune adjuvant able to mimic, enhance, stimulate, and interact with the host immune system especially at the level of DCs. Although PLGA’s immune effects have been studied in some details, other biomaterials used to produce NP may have different chemical properties that affect immune cells. Given the considerable variety of biomaterials that can be used to design NPs, further investigations that aim at identifying the immune stimulant abilities of NP’s components are required. This could be very critical to develop personalized nanomedicine that aims to induce anti-tumor immunity in a predictable and desirable fashion. Similar to the immune system itself, nanodevices present tremendous flexibility and plasticity and could be therefore considered as an IRM platform capable to be tailored according to the desired application. Their unique abilities to encapsulate a high payload of radionuclide; notably high-LET α-particles and low-LET β-emitters; and to undergo surface modifications, further support their strong potential as a new anti-cancer strategy enable to induce effective anti-tumor immunity.

Much remains to be learned about the effect of nanovectorized radiotherapy but initial data showing that the delivery of ionizing radiation via NPs can be effective at inducing anti-tumor immunity suggest that this new approach warrants further investigations.

## Conflict of Interest Statement

The authors declare that the research was conducted in the absence of any commercial or financial relationships that could be construed as a potential conflict of interest.
